# Robot-Assisted Lymph Node-to-Vein Anastomosis: Lessons from the First 22 Cases at a High-Volume Lymphatic Supermicrosurgery Center

**DOI:** 10.3390/curroncol32070377

**Published:** 2025-06-29

**Authors:** Wei F. Chen, David C. F. Cheong, Erica Tedone Clemente, Melis Salman

**Affiliations:** 1 Center for Lymphedema Research and Reconstruction, Department of Plastic Surgery, Cleveland Clinic, Cleveland, OH 44124, USA; 2 Division of Reconstructive Microsurgery, Department of Plastic and Reconstructive Surgery, Chang Gung Memorial Hospital, Linkou Medical Center, Taoyuan 333, Taiwan

**Keywords:** robotic surgery, lymphedema, LNVA, lymphatic reconstruction, supermicrosurgery, cancer survivorship, brain lymphatics

## Abstract

(1) Background: Lymphedema is a common but underrecognized sequela of cancer treatment. Supermicrosurgical procedures such as lymphaticovenular anastomosis (LVA) and, more recently, lymph node-to-vein anastomosis (LNVA) have emerged as effective options for fluid-predominant disease. In 2024, we began performing robot-assisted LNVA using a next-generation microsurgical robot. This study describes our initial experience, technical insights, and the potential for robotics to extend the boundaries of supermicrosurgery. (2) Methods: Twenty-two consecutive robotic LNVAs were performed by a high-volume supermicrosurgeon at a tertiary center. Preoperative imaging with standard and ultra-high frequency ultrasound was used to identify optimal lymph nodes and veins. Robotic LNVA was performed using the Symani Surgical System, with adaptations for motion scaling, ergonomics, and console control. Intraoperative patency was confirmed by direct washout and/or indocyanine green (ICG) transit. (3) Results: All 22 procedures were technically successful, with 100% intraoperative patency. Anastomosis time improved from 37 to 18 min. Robotic assistance enhanced precision, eliminated tremors, and reduced the technical burden of operating at extreme submillimeter scales. (4) Conclusions: Robotic LNVA is safe, feasible, and efficient. It optimizes current techniques, offering the potential to extend surgical access below the 0.1 mm threshold, with implications for future treatment of lymphatic and possibly intracranial disease.

## Simple Summary

Lymphedema is a common complication of cancer treatment, often resulting from unavoidable damage to the lymphatic system. Supermicrosurgical procedures, particularly lymph node-to-vein anastomosis (LNVA), can restore lymphatic drainage, but their technical demands limit the number of surgeons qualified to perform them. As a result, many patients lack access to effective surgical care. Our study reports the first 22 cases of robot-assisted LNVA using an advanced microsurgical robot. The system improved precision, reduced fatigue, and made complex tasks more consistent. Crucially, robotic assistance flattens the steep learning curve of supermicrosurgery, democratizing the procedure and enabling more surgeons to adopt it. All procedures in our series were successful, with operative times improving rapidly with experience. These findings suggest that robotic platforms may not only enhance existing surgical techniques but also unlock future capabilities, potentially allowing access to anatomical structures previously considered too small to treat, such as the lymphatic vessels of the brain.

## 1. Introduction

Lymphedema is a common and often underrecognized sequela of cancer treatment. Surgical lymphadenectomy, radiation therapy, and other oncologic interventions frequently result in iatrogenic lymphatic injury, placing patients at lifelong risk of developing lymphedema. Despite this, reported incidences in the literature significantly underestimate the true prevalence of the disease. This underreporting is largely attributable to an incomplete understanding of the lymphatic disease process and limitations in current diagnostic paradigms. From the moment lymphatic injury occurs, patients enter International Society of Lymphology (ISL) Stage 0, also known as subclinical or asymptomatic lymphedema [1]. This is a pathologic state that often progresses silently until overt symptoms develop.

Over the past two decades, supermicrosurgical interventions, defined as procedures involving structures between 0.1 and 0.8 mm in diameter [2], such as lymphaticovenular anastomosis (LVA), have emerged as effective strategies to restore lymphatic drainage and mitigate disease progression, particularly when performed early. A related concept, lymph node-to-vein anastomosis (LNVA), was originally described by Nielubowicz and Olszewski in 1968 as a microsurgical bypass using viable lymph nodes to divert lymphatic fluid into the venous system [3,4]. By today’s supermicrosurgical standards, the original technique was unnecessarily traumatic to the recruited lymph nodes, which likely contributed to its limited efficacy and inconsistent results.

In 2021, Pak introduced modifications to the procedure that improved surgical outcomes and renewed interest in LNVA [5]. In 2022, our center implemented further refinements aimed at enhancing both the precision and efficiency of the technique [6]. These included preoperative evaluation using ultra-high-frequency (UHF) ultrasound to identify high-flow functional lymph nodes, and intraoperative radar-based tracking to ensure accurate localization. This approach enabled a streamlined, minimally invasive procedure with a high rate of technical success and favorable clinical outcomes. Conceptually, LNVA builds upon the principles of LVA. While LVA connects individual lymphatic vessels to microscopic veins, LNVA recruits the full complement of lymphatic vessels within a lymphosome [7,8], drained by individual lymph nodes, as physiologic conduits into the venous system. Internally, we refer to LNVA as “LVA 2.0” to reflect its expanded physiologic potential.

In 2024, we further advanced our surgical platform by incorporating a next generation microsurgical robot (Symani MMI, Jacksonville, FL, USA) (Figure S1) to perform all supermicrosurgical procedures, including LVA and LNVA. Robotic platforms offer several theoretical advantages in this context, including motion scaling, tremor filtration, and improved ergonomics [9,10,11,12]. However, the transition from a manual to robotic technique introduced critical differences in surgical experience, intraoperative workflow, and performance, particularly from the perspective of a high-volume manual supermicrosurgeon.

In this perspective, we describe our initial experience with 22 cases of robot-assisted LNVA and share the key insights gained during this early phase of adoption. These lessons may promote the best practices, guide future adopters, and help define the evolving role of robotics in the field of lymphatic supermicrosurgery.

## 2. Materials and Methods

### 2.1. Patient Evaluation and Selection for LNVA

All patients undergo a comprehensive history and physical examination at intake. The history focuses on distinguishing between primary and secondary lymphedema, as well as characterizing the disease as fluid predominant, solid predominant, or mixed. A history of lymphatic injury, such as wide local excision or lymphadenectomy, suggests acquired (secondary) disease. Reports of poor or absent responsiveness to compression therapy, particularly when accompanied by irreversible or partially reversible limb bulk, are suggestive of solid predominant or mixed disease, respectively.

Diagnosis of lymphedema and characterization of its fluid or solid components are further corroborated using indocyanine green lymphography [13], bioimpedance spectroscopy with body composition analysis [14,15,16], and three-dimensional volumetric scanning [17,18].

LVA and LNVA are indicated only for fluid-predominant lymphedema, as neither procedure effectively addresses the solid components associated with lymphedema-induced lipodystrophy or fibrosis [6,19]. Preoperative surveillance imaging with standard ultrasound (less than 15 MHz) is performed to evaluate for the presence of suitable lymph nodes. Once candidate nodes are identified, UHF ultrasound (22 and 46 MHz) is used to select the healthiest and most functional lymph node as the surgical target, based on high-resolution sonographic features [20,21]. The selected lymph node is then marked with an 8 mm nonradioactive Savi Scout^®^ radar reflector (Merit Medical, South Jordan, Utah) placed under ultrasound guidance [22]. For patients with arm lymphedema, epitrochlear and brachial lymph nodes are assessed. For leg lymphedema, inguinal and popliteal nodes are targeted. During the ultrasound examination, the nearby vein targets for LNVA are also identified and marked. For arms, branches of cephalic and basilic veins are used. For legs, superficial circumflex iliac vein, superficial inferior epigastric vein, and superficial external pudendal vein are used in the groin, and popliteal and Giacomini vein are used in the popliteal fossa (Table 1). When suitable lymph nodes are available, LNVA is preferentially considered over LVA due to its observed superior efficacy in our experience. In cases where no usable nodes are visualized, LVA remains the procedure of choice.

### 2.2. Robotic LNVA: Surgical Technique

**Figure 1:**
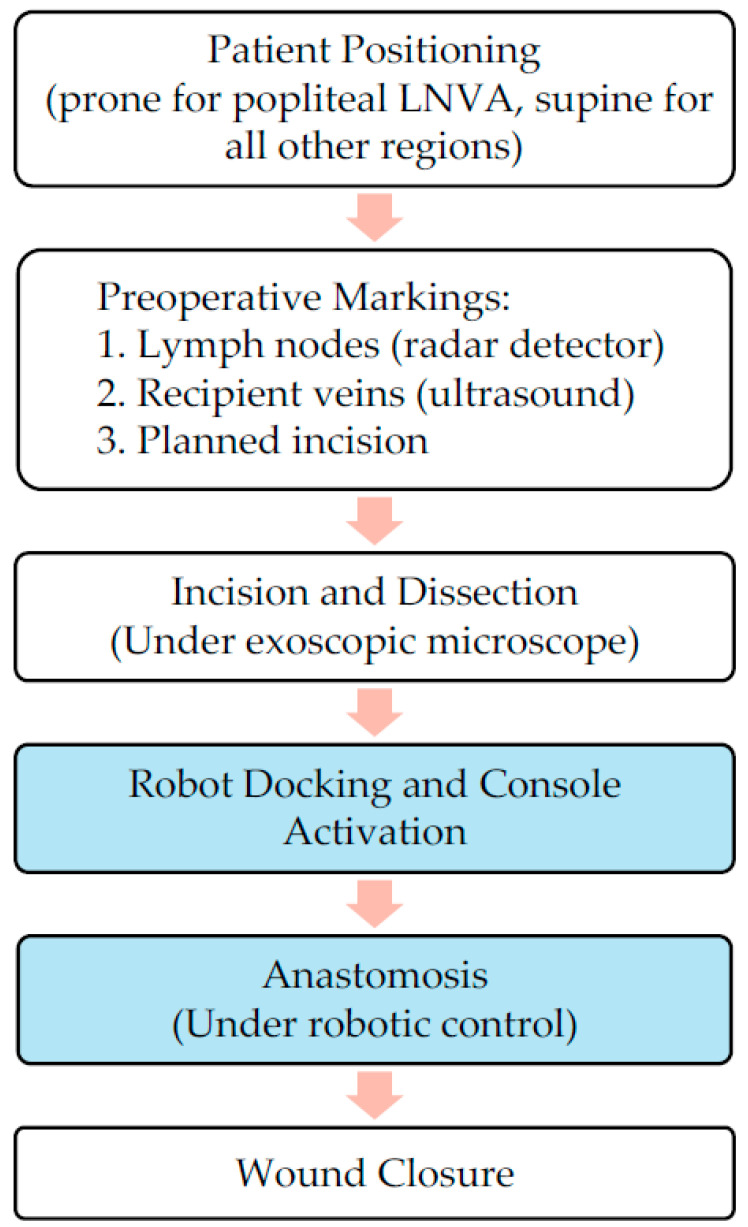
Overview of robot-assisted LNVA workflow. (Blue-highlighted boxes indicate the steps involving robotic assistance).

**Figure 2:**
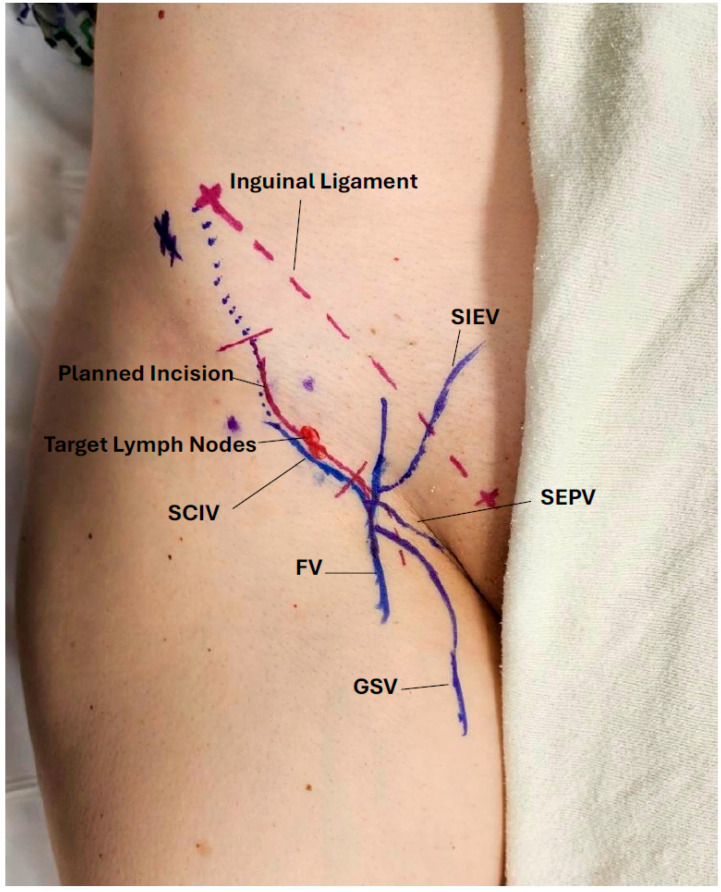
Preoperative markings of lymph nodes, recipient veins, and planned incision. FV: Femoral vein; GSV: Greater saphenous vein; SCIV: Superficial circumflex iliac vein; SEPV: Superficial external pudenal vein; SIEV: Superficial inferior epigastric vein.

The overview of robotic-assisted LNVA workflow is illustrated in Figure 1. Patients are positioned supine for all procedures except popliteal LNVA, which requires a prone position. For upper extremity cases, the arm is extended perpendicularly from the torso and supported on an arm table. Following induction of general anesthesia, a curvilinear skin incision is designed to allow optimal exposure of both the target lymph node and vein (Figure 2).

The procedure is performed using a 4K ultra-high-definition, three-dimensional video exoscopic surgical microscope (Mitaka, Tokyo, Japan), which offers up to 110× magnification. The operative field is visualized on a 75-inch monitor with stereoscopic 3D enhancement. We prefer the exoscope setup due to its compact optical unit, which minimizes interference with the robotic arms and avoids the “sword fighting” often encountered with conventional surgical microscopes.

**Figure 3:**
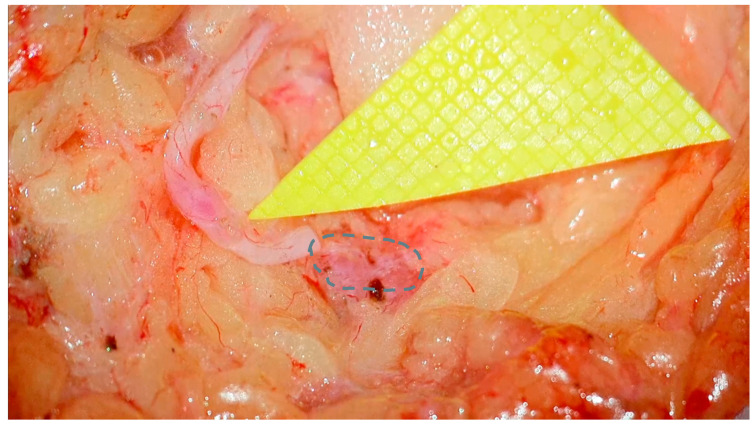
Target lymph node (dotted line) and vein prepared for anastomosis. The yellow triangular standard microsurgery background features 1 mm grid squares, providing a visual reference for scale.

After the skin incision, meticulous dissection is carried out to isolate the pre-identified lymph node and vein. This step is efficient due to prior localization. Intraoperative use of the Savi Scout^®^ probe aids in node identification by producing increasingly rapid auditory signals as the probe approaches the radar-marked lymph node. Once both the lymph node and vein are exposed, they are prepared for anastomosis. The anterior capsule of the lymph node is cleared of surrounding areolar tissue to create a suitable landing zone, with care taken to preserve afferent and efferent lymphatic vessels, as well as hilar arterioles and venules. A sufficient length of the target vein is dissected to ensure a tension free anastomosis. After confirming optimal vein orientation and axiality, the vein is ligated and brought adjacent to the target node (Figure 3).

**Figure 4:**
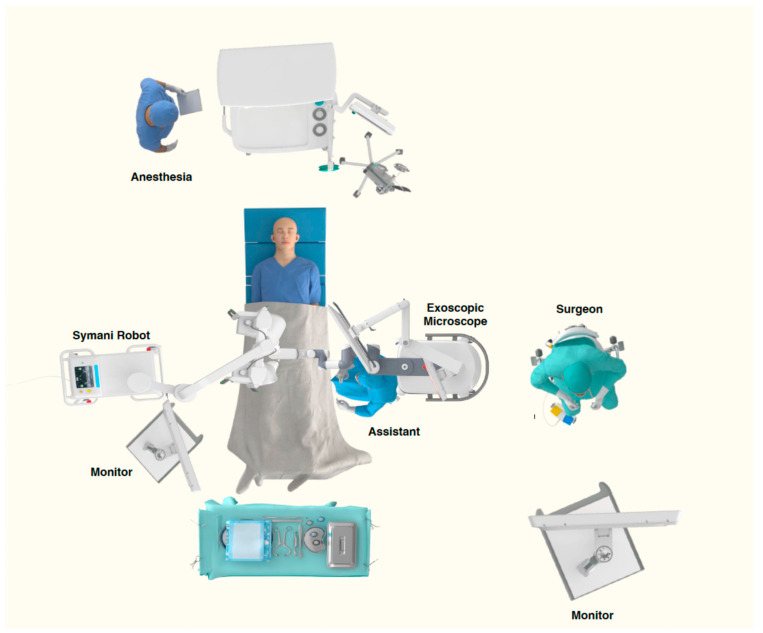
The robot and microscope position settings for lymph node-to-vein anastomosis (LNVA) of right groin. *Image modified from original image provided by MMI (Medical Microinstruments, Inc., Jacksonville, FL, USA) with permission.*

Robotic assistance begins at this point. The surgeon leaves the operative field and assumes position at the robotic console. The robotic system (Symani Surgical System, MMI, Jacksonville, FL, USA) is brought into position, and its paired arms are docked (Figure 4). The surgeon then operates the robot using free-floating, chopstick-like controllers. An 11-0 suture with an 80-micrometer needle is preferred, as lymph nodes affected by lymphedema often have a thickened fibrotic capsule. The robotic supermicrosurgical anastomosis is completed with interrupted sutures, similar to manual technique (Video S1). Maneuvers such as back wall first suturing, continuous loop technique, and airborne sutures may be employed to address challenging anatomy, as in manual anastomosis. Following completion of the anastomosis, the surgical field is irrigated and hemostasis confirmed. Wound closure is performed manually using standard layered techniques.

## 3. Results: Insights from Our Early Robotic-LNVA Experience

### 3.1. Initial Impressions, Learning Curve, and Surgical Outcomes

All procedures in this series were performed by the senior author (WFC). From the outset, the robotic system proved to be highly intuitive. The fidelity with which the robotic arms replicated manual hand movements was remarkable, and the built-in tremor elimination feature effectively neutralized physiological hand tremor. The translation of large-scale motions into precise supermicrosurgical maneuvers through movement scaling (ranging from 7 to 20 times) introduced an interesting but manageable challenge. Although the free-floating controllers provided a natural interface, their use required a brief initial adjustment period. In contrast to manual supermicrosurgery, which relies primarily on fine motor control at the interphalangeal and metacarpophalangeal joints, robotic supermicrosurgery shifts the burden to larger joints, including the wrist, elbow, and occasionally the shoulder, due to the need for broader movements to generate scaled operative actions. This shift in motor effort, particularly at higher scaling ratios, was initially the most unfamiliar aspect of robotic control. However, with experience, this discomfort quickly subsided, and the advantages of enhanced precision became readily apparent.

Proficiency improved rapidly. Anastomosis time decreased from 37 min in the 1st case to 18 min by the 22nd case, closely approaching our average manual LNVA time of 15.7 min. Although the limited case volume precludes formal modeling of a complete learning curve, we subjectively observed the most substantial technical progress within the first eight cases. Beyond that point, further improvements were noted but became increasingly incremental and less perceptible. In this series, we achieved 100 percent (22 of 22) patency of the robotic-assisted anastomoses. Patency was confirmed intraoperatively by direct visualization of the washout sign and/or by successful passage of indocyanine green dye from the lymph node into the vein.

### 3.2. Technical Tips and Pearls

Based on our early robotic-LNVA experience, we offer the following technical considerations to optimize surgical efficiency and precision:

1. **Adjust Movement Scaling Based on Task Requirements**

While movement scaling enhances surgical precision, it also increases the physical range of motion required from the surgeon. A higher scaling ratio, such as 10-to-1, demands broader hand movements but provides greater robotic assistance. In contrast, a lower ratio, such as 7-to-1, requires less movement but offers more direct manual control. We found it optimal to tailor the scaling ratio to the anastomosis size—using 10-to-1 for vessels smaller than 0.5 mm and 7-to-1 for larger targets. In our experience, scaling beyond 10-to-1 has not been necessary for LNVA. However, higher scaling ratios may be beneficial for surgeons who are earlier in their supermicrosurgical training or who would benefit from additional stabilization support.

2. **Coordinate Arm Support with Movement Scaling**

**Figure 5:**
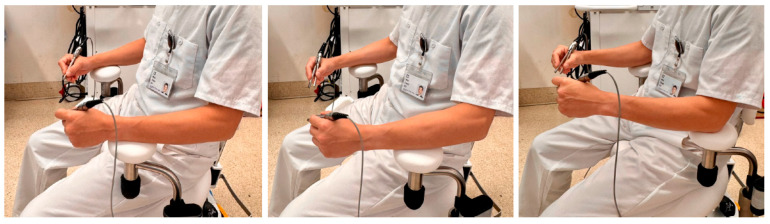
(**Left**, **middle**) Supporting the wrist and forearm at lower scaling ratio (7×); (**right**) supporting the elbow at higher scaling ratio (10× or higher). The surgeon should move the support more proximally when larger movements are expected (higher scaling), and more distally when less movements are needed (lower scaling).

Arm support positioning should correspond to the range of motion dictated by the selected scaling ratio. When using lower scaling ratios such as 7×, only finger/hand movements are needed, and placing the support under the forearm increases stability by restricting elbow motion. In contrast, at higher scaling ratios such as 10×, elbow mobility becomes necessary, and the support should be positioned directly under the elbow to allow full joint movement. We recommend determining the desired scaling ratio first, then setting arm support accordingly to optimize ergonomics (Figure 5).

3. **Adapt Instrument Handling to Electromagnetic Field Constraints**

The robotic system is controlled via free-floating handheld controllers, which must remain within a defined electromagnetic field (70 × 50 × 45 cm) projected from the surgeon’s console (Figure S2). If either controller exits this field, the system pauses and requires re-engagement. At higher scaling ratios, broader hand movements increase the likelihood of unintentionally exiting the field. To accommodate this, standard single-hand techniques, such as one-handed suture pulling, should be modified. We recommend using a two-hand, two-point pull technique (Video S1), which allows tensioning of the suture while keeping both controllers within the active field. Similar adaptations may be necessary for other tasks, favoring coordinated bimanual techniques over unilateral maneuvers.

4. **Caffeine Consumption**

We have found that robotic assistance largely neutralizes the impact of physiological hand tremors, including that induced by caffeine. The senior author, who traditionally adjusted his caffeine intake prior to manual supermicrosurgery, was pleased to discover that with robotic support, such adjustments were no longer necessary. Full-function performance was maintained even with a robust caffeine routine. While not a formal performance metric, the ability to operate with steady precision after one’s usual morning coffee was a welcomed and practical advantage.

## 4. Discussion

### 4.1. Unexpected Observations and Surgical Insights

Modern supermicrosurgery enables access to structures ranging from 0.8 mm down to 0.1 mm in diameter. To our knowledge, only a few surgeons are capable of consistently operating at the 0.1 mm level, and none have been able to reliably operate below this threshold using manual techniques. As the caliber of the target structure decreases, surgeons experience a clear escalation in demands on hand control, fine motor dexterity, instrument stability, and sustained mental focus, particularly during sub-0.4 mm anastomoses.

One of the most striking observations in our robotic-LNVA experience was how the robot mitigated these escalating technical and cognitive demands. With robotic assistance, the perceived difference in difficulty between anastomosing a 0.8 mm vessel and a 0.1 mm vessel was substantially diminished. What once felt like distinct technical milestones across the submillimeter scale, such as transitioning from 0.8 mm to 0.4 mm, and then to 0.1 mm, now feels remarkably similar under robotic control. In essence, the robot flattens the technical curve of extreme supermicrosurgery.

This observation carries meaningful implications. If robotic platforms can overcome the manual barriers associated with the 0.1 mm threshold, the current practical lower limit of human-performed surgery, it may become feasible to push beyond this boundary. The advent of robotic supermicrosurgery may not only expand what is technically achievable, but also redefine the fundamental limits of human surgical precision.

### 4.2. The Future of Robotic Supermicrosurgery

Our early experience with robot-assisted LNVA highlights the potential of robotic technology to advance the field of extreme precision surgery. The system’s intuitive control, motion scaling, and tremor elimination, enabled rapid adoption and improved accuracy, even at the smallest operative scales.

Beyond refining current techniques, the robot presents the possibility of pushing supermicrosurgery beyond its current lower limit of 0.1 mm. This threshold has historically defined what is surgically accessible and has enabled transformative treatments for lymphedema, once considered untreatable and now, in some cases, curable. Going smaller may unlock even greater possibilities.

One particularly intriguing frontier is the brain’s lymphatic system. Promising supermicrosurgical treatments already exist for brain lymphatic dysfunction, but these rely on extracranial interventions that influence the intracranial glymphatic and meningeal systems indirectly. Direct manipulation of the brain’s lymphatics has long remained out of reach due to their sub-0.1 mm caliber. Robotic platforms may finally bring these delicate structures within surgical range. Whether direct intervention will prove more effective than extracranial approaches remains unknown, but for the first time, it appears technically possible.

## 5. Conclusions

Robotic assistance has redefined what is surgically possible in the supermicrosurgical domain. Our early experience with robot-assisted LNVA demonstrated not only technical feasibility but also meaningful advantages in precision, consistency, and ergonomics. As this technology evolves, it holds the potential to push surgery beyond the current 0.1 mm frontier. With it, we may one day access and directly treat anatomical structures that have remained beyond the reach of human hands, perhaps even the brain’s lymphatics. What once limited us physically may soon be limited only by imagination.

## Figures and Tables

**Table 1:** Target lymph nodes and veins for LNVA by region.

Region	Target Lymph Nodes	Target Veins
arm (upper extremity)	epitrochlear and brachial	branches of cephalic and basilic veins
groin (upper leg)	inguinal	superficial circumflex iliac, superficial inferior epigastric, and superficial external pudendal veins
popliteal fossa (lower leg)	popliteal	popliteal vein and Giacomini vein

## Data Availability

The data supporting the findings of this study are not publicly available due to privacy and ethical restrictions.

## Supplementary Materials

The following supporting information can be downloaded at: https://www.mdpi.com/article/10.3390/curroncol32070377/s1; Figure S1: Three main components of the robot, Symani^®^ Surgical System.; Figure S2: The surgeon console consists of five units. The surgeon sits on the ergonomic chair (1) and uses the controllers (2) within a workspace (orange box), detected by the tracking unit (3). The robot can be controlled via footpedal (4) when the system cable connector (5) is connected to the CMM (Cart, Macropositioner, Micromanipulators); Video S1: Robotic LNVA.
