# Supplementary material for: Robot-Assisted Lymph Node-to-Vein Anastomosis: Lessons from the First 22 Cases at a High-Volume Lymphatic Supermicrosurgery Center
Source: Curr Oncol. 2025 Jun 29;32(7):377. doi: 10.3390/curroncol32070377 (PMC12293415; doi:10.3390/curroncol32070377)

Figure S1. Three main components of the robot, Symani® Surgical System. *Image modified from original image provided by MMI (Medical Microinstruments, Inc.) with permission.*

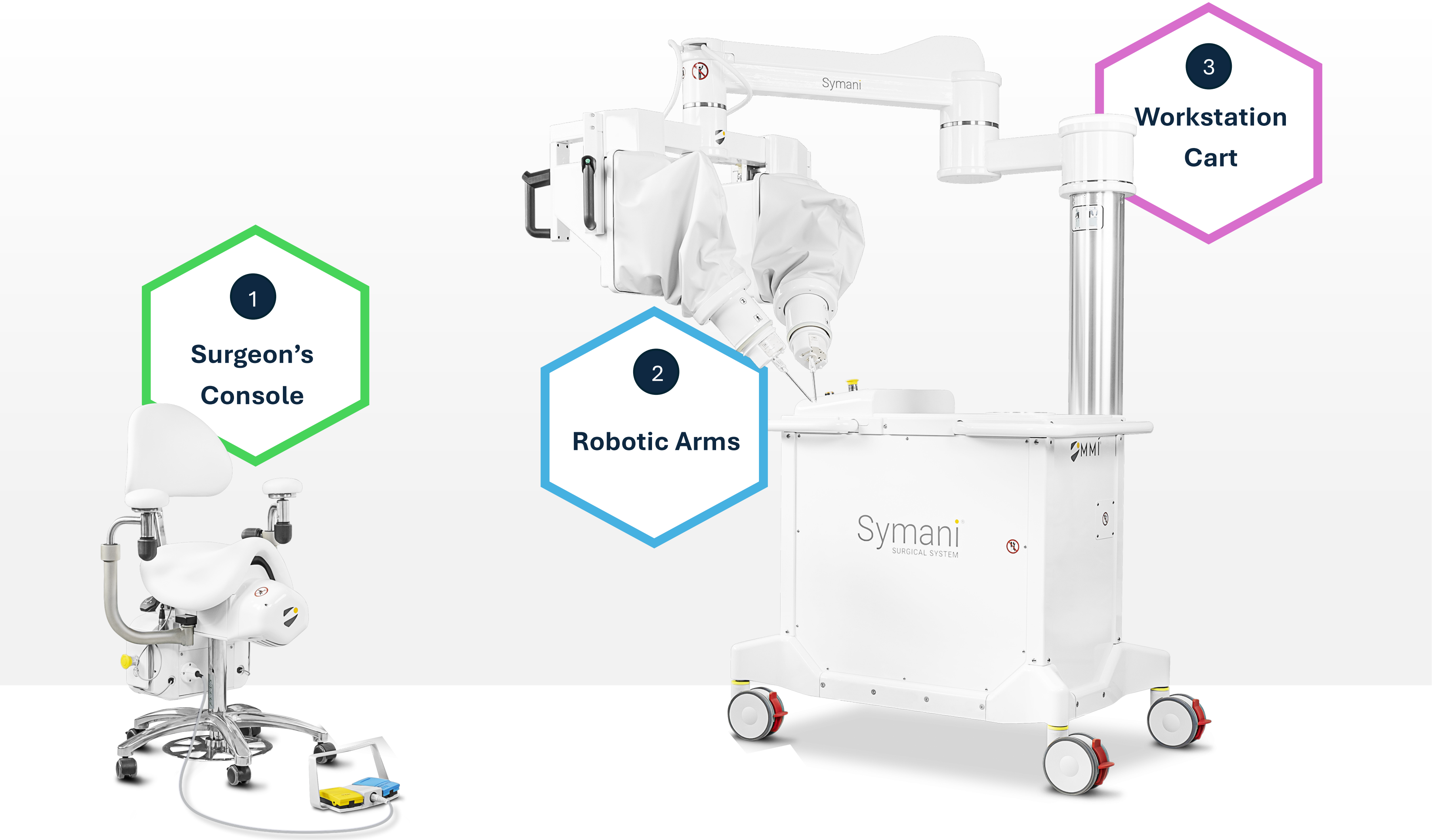

Supplement: Supplementary file 1 [file curroncol-32-00377-s001.zip › Figure S1. Three main components of the robot.pdf]
