# Supplementary material for: Robot-Assisted Lymph Node-to-Vein Anastomosis: Lessons from the First 22 Cases at a High-Volume Lymphatic Supermicrosurgery Center
Source: Curr Oncol. 2025 Jun 29;32(7):377. doi: 10.3390/curroncol32070377 (PMC12293415; doi:10.3390/curroncol32070377)

Figure S2. The surgeon console consists of 5 units. The surgeon sits on the ergonomic chair (1) and uses the controllers (2) within a workspace (orange box), detected by the tracking unit (3). The robot can be controlled via footpedal (4) when the system cable connector (5) is connected to the CMM (Cart, Macropositioner, Micromanipulators, not shown). *Image modified from original image provided by MMI (Medical Microinstruments, Inc.) with permission.*

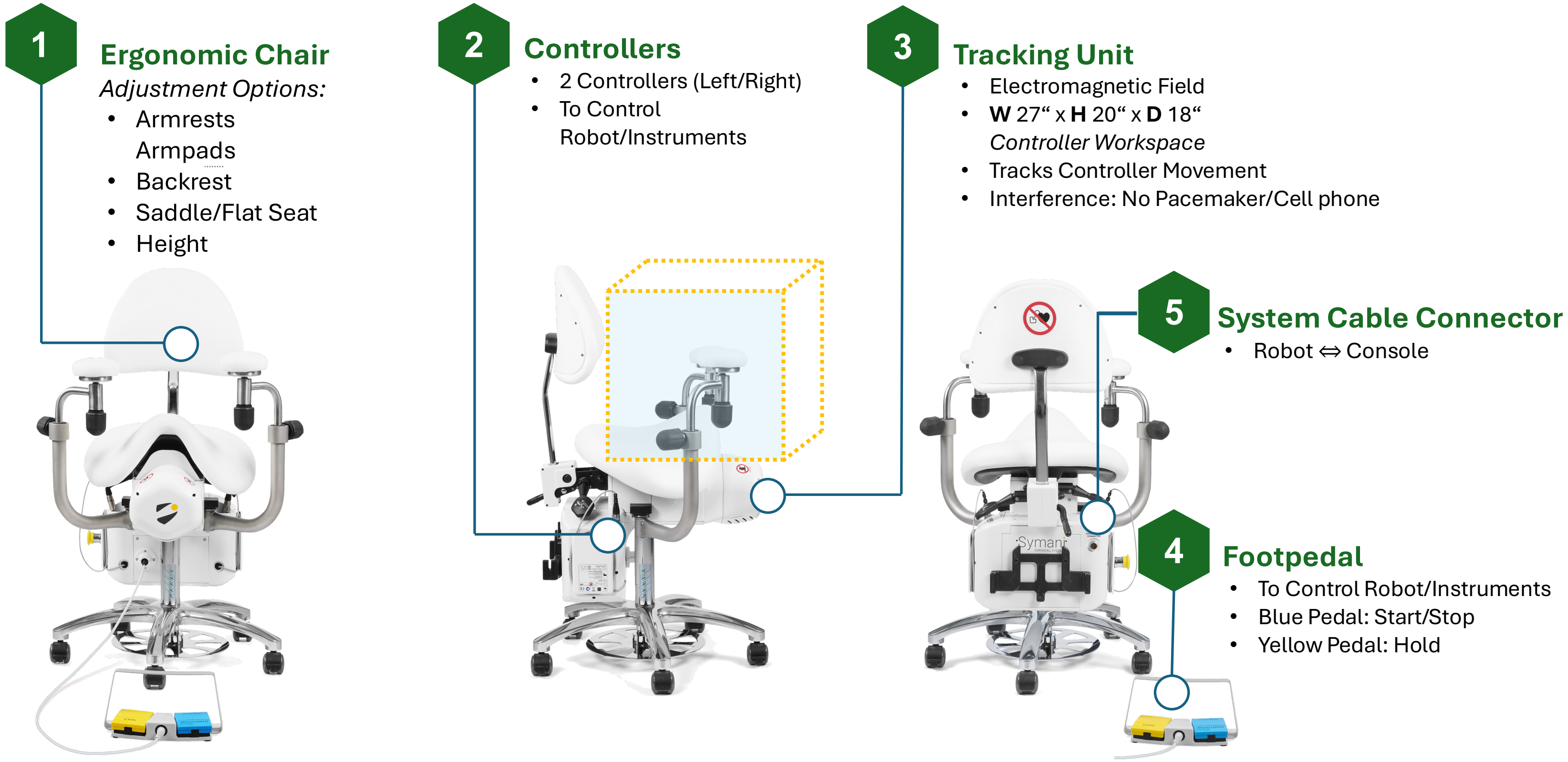

Supplement: Supplementary file 1 [file curroncol-32-00377-s001.zip › Figure S2. Surgeon Console.pdf]
